# The Promotion of Puerperal Fever

**Published:** 1887-11

**Authors:** 


### *THE PROMOTION OF PUERPERAL FEVER.*

Dr. W. S. Playfair read, before the British Medical Association, a paper on the promotion of puerperal fever. In it, he made a statement concerning the general practitioners of England, which we think applies as well to those of America. "I doubt if, even yet, antiseptic midwifery is at all general in private practice." He believes that the monthly nurses, especially the older class, consider the antiseptic precautions as "new-fangled fads," and follow but the physician's directions only in the most perfunctory way. He, therefore, has a printed card which he gives to every nurse in attendance upon a case under his charge. We give a copy of it below. It may give many an idea which they will find profitable to put into practice :

### ANTISEPTIC RULES FOR MONTHLY NURSES.

"1. Two bottles are supplied to each patient. One contains a solution of perchloride of mercury, of the strength of one part to 1,000 of water, tinted with litmus (called the one to 1,000 solution), the other, carbolized oil (one to eight).

"2. A small basin containing the one to 1,000 solution must always stand by the bedside of the patient, and the nurse must thoroughly rinse her hands in it every time she touches the patient in the neighborhood of the genital organs, for washing or any other purpose, before or during labor, or for a wash after delivery.

"3. All sponges, vaginal and rectal pipes, catheters, etc., must be dipped in the one to 1,000 solution before being used. The surface of slippers, bed-pan, etc., should also be sponged with it.

"4. Vaginal pipes, enema tubes, catheters, etc., should be smeared with carbolized oil before use.

"5. Unless express directions are given to the contrary, the vagina should be sponged daily after delivery with warm water, with a sufficient quantity of Coudy's fluid dropped into it to give a pale pink color.

"6. All soiled linen, diapers, etc., should be immediately removed from the bed-room.

"N. B.—These rules are for the protection of the patient from the risk arising from accidental contamination of the hands, sponges, etc. It is, therefore, hoped that they will be faithfully and minutely adhered to."

These rules as they are, or varied to suit the ideas of the physician or the form of antiseptic used, might do a great deal to enlighten the many nurses in America who are totally ignorant of antiseptics.

